# Subclinical cardiac dysfunction in idiopathic inflammatory myopathies: the role of global longitudinal strain

**DOI:** 10.1007/s10238-026-02054-1

**Published:** 2026-02-17

**Authors:** Simone Romano, Andrea Sartorio, Chiara Dal Pont, Francesca Segatta, Marta Piazzola, Marco Vicardi, Mattia Cominacini, Federico Aldegheri, Riccardo Bixio, Ombretta Viapiana

**Affiliations:** 1 Department of Internal Medicine, Section of Internal Medicine C, University of Verona, Piazzale Scuro 37134, Verona, Italy; 2 Rheumatology Unit, University of Verona, Verona, Italy

**Keywords:** Idiopathic inflammatory myopathies, Echocardiography, Global longitudinal strain, Arthritis

## Abstract

Autoimmune diseases are characterized by systemic inflammation that can affect multiple tissues. In idiopathic inflammatory myopathies (IIM), skeletal muscle is primarily involved; however, subclinical cardiac dysfunction may also occur. While left ventricular ejection fraction (EF) is commonly used to assess cardiac function, global longitudinal strain (GLS) has proven more sensitive in detecting early myocardial impairment. This study aimed to evaluate left ventricular GLS (LV GLS) in patients with IIM and no known cardiovascular disease, assessing both the prevalence of reduced GLS values and their associations with clinical and laboratory parameters. We enrolled 37 outpatients from the Department of Internal Medicine at the University Hospital of Verona, who underwent comprehensive clinical and echocardiographic assessment. The mean GLS value observed (− 17.9% ± 2.2%) was below the normal reference range (− 18.2% to − 21.2%) defined by the echocardiographic system. When compared with 37 healthy controls, IIM patients showed significantly impaired GLS despite preserved EF in both groups (− 17.88 ± 2.23% vs. − 19.88 ± 1.72%, *p* < 0.001). This difference remained significant after adjusting for age and sex (β = +2.25%, *p* < 0.001). In linear regression models, GLS was independently associated with arthritis (β = 2.165, *p* = 0.007), lymphocyte count (β = 0.001, *p* = 0.025), CK levels (β = 0.0003, *p* = 0.024). Patients with arthritis showed significantly worse GLS values compared to those without arthritis, despite similar EF. In multivariate analysis, arthritis remained independently associated with impaired GLS and lower CK levels. Overall, our findings suggest that patients with IIM exhibit a global reduction in left ventricular longitudinal function, detectable by GLS, even in the absence of overt cardiac disease. This impairment appears particularly evident in patients presenting with arthritis and is independent of age-related effects. Longitudinal studies are warranted to investigate the progression of GLS alterations and their potential role in guiding therapeutic strategies.

## Introduction

Idiopathic inflammatory myopathies (IIM) are a heterogeneous group of autoimmune diseases primarily characterized by chronic inflammation of skeletal muscle. However, due to their systemic nature, these disorders can also affect extramuscular organs, including the heart. Cardiac involvement in IIM is frequently subclinical and may remain undetected, yet it is associated with increased morbidity and mortality [1].

Left ventricular ejection fraction (EF) has traditionally been the standard echocardiographic parameter used to assess cardiac function. Although widely applied, EF is relatively insensitive to early myocardial dysfunction, particularly when the impairment affects longitudinal fibers of the left ventricle. In recent years, global longitudinal strain (GLS) has emerged as a more sensitive and reproducible echocardiographic marker for detecting early myocardial impairment, even in patients with preserved EF [2–8].

Several studies have demonstrated that patients with autoimmune diseases may exhibit reduced GLS values, indicating early subclinical myocardial involvement [9–13]. This is particularly relevant in the context of IIM, where cardiac symptoms are often absent or overshadowed by dominant muscular manifestations. GLS, therefore, may serve as a valuable tool for detecting cardiac dysfunction in its subclinical phase [14–16].

The aim of this study was to evaluate left ventricular GLS (LV GLS) in a cohort of patients with IIM and no known cardiovascular disease. Additionally, we sought to investigate the associations between GLS values and clinical or laboratory parameters, in order to identify potential predictors of myocardial dysfunction in this population.

## Methods

### Study design and participants

We conducted a retrospective observational study involving 37 adult outpatients diagnosed with IIM at the Department of Internal Medicine, University Hospital of Verona. All patients had preserved LVEF and no prior history or clinical evidence of cardiovascular disease. Clinical, demographic, and laboratory data were collected during the outpatient visit and by reviewing the hospital’s electronic medical records.

Inclusion criteria: Adult patients (≥ 18 years) with a diagnosis of IIM according to Bohan and Peter criteria or 2017 EULAR/ACR classification criteria, with available echocardiographic data including speckle-tracking analysis. Exclusion criteria: known cardiovascular disease (coronary artery disease, heart failure, significant valvular disease), atrial fibrillation.

### Control group selection

The control group was retrospectively identified from the institutional echocardiographic database. Controls were selected among subjects who underwent transthoracic echocardiography for non-cardiac indications (such as exclusion of structural heart disease) and who showed normal cardiac structure and function, preserved left ventricular ejection fraction, and no history of cardiovascular disease. Subjects with ischemic heart disease, cardiomyopathy, significant valvular disease, atrial fibrillation, or other relevant arrhythmias were excluded.

### Clinical definitions

Interstitial lung disease (ILD) was defined by the presence of HRCT findings consistent with interstitial pneumonia (e.g., NSIP, UIP, or organizing pneumonia patterns) as reported by the radiologist. Arthritis was defined as clinically evident joint swelling and tenderness documented by the rheumatologist. Cutaneous involvement included typical dermatomyositis-related manifestations such as heliotrope rash, Gottron’s papules, or extensor surface erythema, when described in the clinical records. Other clinical features (e.g., Raynaud phenomenon or dysphagia) were extracted directly from specialist documentation. Due to the retrospective design, definitions reflected routine clinical practice rather than formal classification criteria.

Laboratory values including creatine kinase (CK), C-reactive protein (CRP), and lymphocyte count were recorded at disease onset when available. Autoantibody testing and myositis-specific antibodies were evaluated when clinically indicated.

### Echocardiographic assessment

**Fig. 1:**
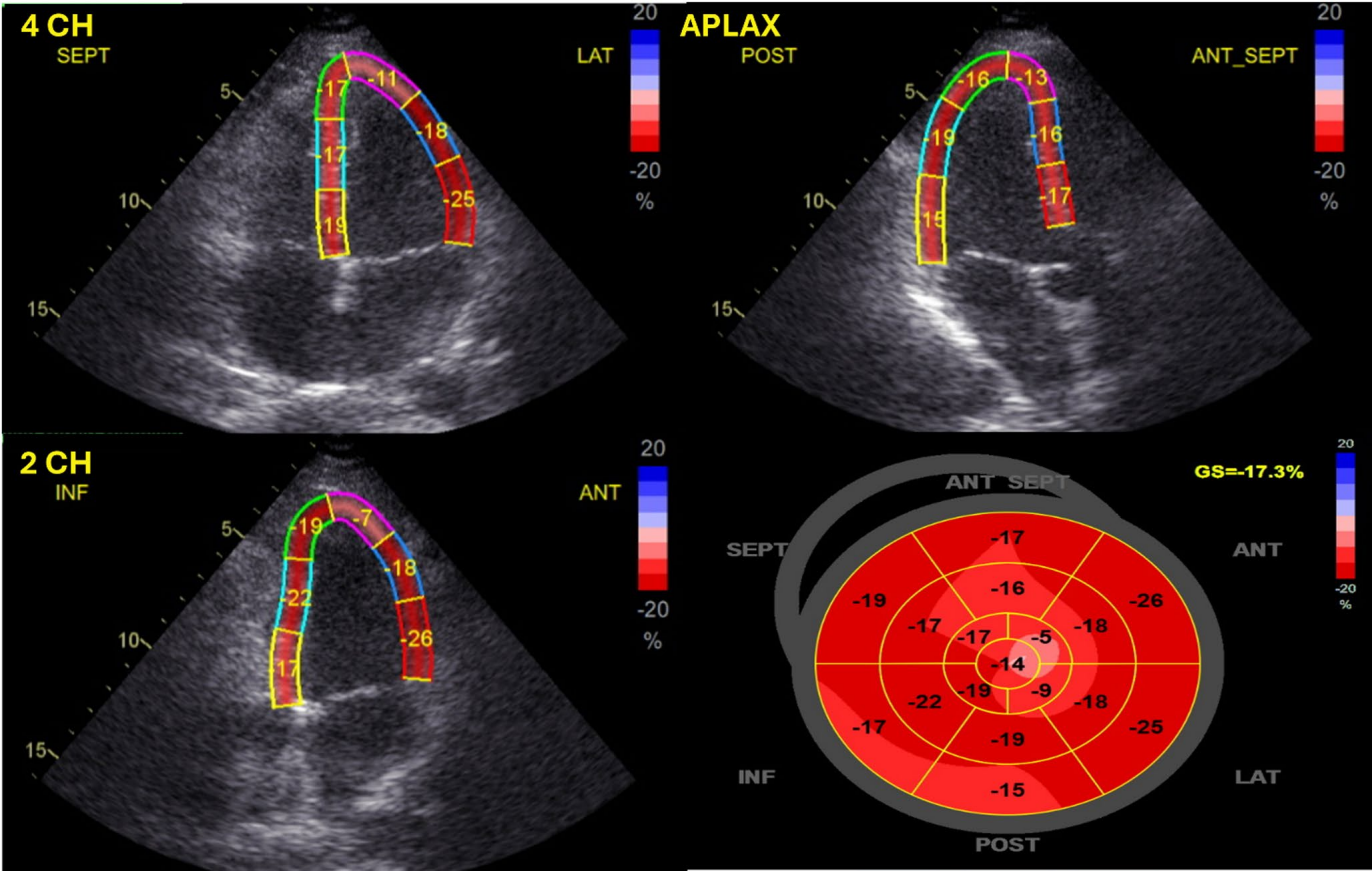
Speckle-tracking echocardiographic strain analysis of a representative patient from the study cohort. Apical four-chamber (4CH), three-chamber (APLAX), two-chamber (2CH), views are presented, each displaying regional peak systolic longitudinal strain. The bull’s-eye plot integrates data from all 18 LV segments derived from these views and provides the global longitudinal strain (GLS) value. In this case, GLS is reduced, reflecting subclinical left ventricular systolic dysfunction

Transthoracic echocardiography was performed using a GE Vivid T8 ultrasound system (GE Healthcare, Arlington Heights, IL). Reference values for GLS ranged from − 18.2% to − 21.2%, as provided by the echocardiographic platform. Standard parasternal, apical, and subcostal views were obtained. GLS was measured using speckle-tracking echocardiography. Electrocardiographically gated cine loops were acquired in apical 2-, 3-, and 4-chamber views. GLS was calculated as the average strain across 18 myocardial segments (Fig. 1).

Endocardial borders were automatically delineated at end-diastole. The tracking was visually verified and manually adjusted if necessary. Segments with poor tracking quality were excluded from the analysis. All measurements were performed by a board-certified echocardiographer blinded to the patients’ clinical and laboratory data.

### Statistical analysis

Statistical analyses were conducted using Jamovi software (version 2.3.18, The Jamovi project, 2022). Continuous variables were reported as mean ± standard deviation (SD) or median with interquartile range (IQR), according to their distribution. Group comparisons were performed using the Student’s t-test or the Mann–Whitney U test, as appropriate. Categorical variables were compared using the chi-square test or Fisher’s exact test.

For comparison with controls, a linear regression model was used with GLS as the dependent variable and group status (IIM vs. control) as the main predictor, adjusting for age and sex as covariates. Linear regression models were also used to assess associations between GLS and clinical variables within the IIM group, and logistic regression models were employed to identify predictors of arthritis. A two-tailed p-value < 0.05 was considered statistically significant.

## Results

### Patient characteristics

The cohort included 37 patients with IIM (83.3% female, mean age 64.8 ± 10.7 years). Patient characteristics are summarized in Table 1. The most common clinical manifestations were arthritis (62.2%), skin manifestations (41.7%), interstitial lung disease (40.5%), and dysphagia (21.6%). Laboratory findings at disease onset showed elevated CK levels (median 492 IU/L) and mild inflammatory markers. The cohort included 15 patients with dermatomyositis (41.7%), 11 with anti-synthetase syndrome (30.6%), 6 with polymyositis (16.7%), and 4 with necrotizing or overlap myositis (11.1%). Myositis-specific antibodies were detected in 32/36 patients (88.9%), with anti-Jo1 (30.6%) and anti-Ro52 (33.3%) being the most frequent. Most patients were receiving immunosuppressive therapy, including corticosteroids (88.9%), mycophenolate (72.2%), and rituximab (55.6%). Laboratory and echocardiographic assessments were performed at the same timepoint.

### Echocardiographic findings and comparison with controls

The mean GLS value in the study population was − 17.9% ± 2.2%, below the reference range provided by the echocardiographic system (− 18.2% to − 21.2%). When compared with 37 healthy controls from our database, IIM patients showed significantly impaired GLS despite preserved EF in both groups. Controls were younger (42.2 ± 17.0 vs. 64.8 ± 10.7 years, *p* < 0.001) and had different sex distribution (45.9% vs. 83.3% female), but showed better GLS values (− 19.88 ± 1.72% vs. − 17.88 ± 2.23%, *p* < 0.001). In a linear regression model adjusting for age and sex, IIM patients maintained significantly impaired GLS compared to controls (β = +2.25%, *p* < 0.001), confirming that the observed reduction was independent of demographic differences. When stratified by sex, no significant differences in GLS were observed, although EF was slightly lower in males. Women had significantly lower body weight compared to men (Table 1).

**Table 1:** Patient characteristics of the IIM cohort, including sex-based comparisons

Variable	Total (*n* = 37)	Male (*n* = 7)	Female (*n* = 30)	*p*
**Demographics**				
Age, years, mean (SD)	63.8 (10.6)	61.1 (8.3)	64.4 (11.1)	n.s.
Age of onset, years, mean (SD)	54.9 (11.7)	54.6 (9.2)	54.9 (12.3)	n.s.
Weight, kg, mean (SD)	70.0 (12.2)	83.2 (13.4)	67.3 (10.3)	0.006
Time to therapy, months, median (IQR)	3 (4)	3.5 (2.5)	2 (4)	n.s.
**IIM Subtypes**,** % (n)**				
Dermatomyositis	40.5 (15)	28.6 (2)	43.3 (13)	n.s.
Anti-synthetase syndrome	29.7 (11)	42.9 (3)	26.7 (8)	n.s.
Polymyositis	16.2 (6)	14.3 (1)	16.7 (5)	n.s.
Necrotizing/Overlap myositis	13.5 (5)	14.3 (1)	13.3 (4)	n.s.
**Myositis-Specific Antibodies**,** % (n)**				
Any MSA positive	86.5 (32/37)	85.7 (6/7)	86.7 (26/30)	n.s.
Anti-Jo1	29.7 (11/37)	42.9 (3/7)	26.7 (8/30)	n.s.
Anti-Ro52	32.4 (12/37)	28.6 (2/7)	33.3 (10/30)	n.s.
Anti-Mi2	8.1 (3/37)	0 (0/7)	10.0 (3/30)	n.s.
Anti-SRP	5.4 (2/37)	14.3 (1/7)	3.3 (1/30)	n.s.
Other MSAs	10.8 (4/37)	0 (0/7)	13.3 (4/30)	n.s.
**Laboratory Parameters**				
Lymphocytes at onset, mean (SD) [µL normal range: 1000–4000]	1945 (881)	2370 (1077)	1848 (823)	n.s.
CK at onset, median (IQR) [U/L, normal: F < 145, M < 171]	492 (1112)	843 (789)	461 (1298)	n.s.
CRP at onset, median (IQR) [mg/L, normal range]	3.0 (8.0)	4.0 (2.5)	3.0 (4.0)	n.s.
ANA titer, median (IQR)	160 (480)	320 (0)	160 (640)	n.s.
**Clinical Manifestations**,** % (n)**				
Arthritis	62.2 (23)	42.9 (3)	66.7 (20)	n.s.
Skin manifestations	40.5 (15)	42.9 (3)	40.0 (12)	n.s.
Interstitial lung disease	40.5 (15)	71.4 (5)	33.3 (10)	n.s.
Dysphagia	21.6 (8)	14.3 (1)	23.3 (7)	n.s.
Gastrointestinal symptoms	24.3 (9)	14.3 (1)	26.7 (8)	n.s.
Pulmonary arterial hypertension	2.7 (1)	14.3 (1)	0 (0)	n.s.
**Current Immunosuppressive Therapy**,** % (n)**				
Corticosteroids	89.2 (33)	85.7 (6)	90.0 (27)	n.s.
Mycophenolate mofetil	70.3 (26)	71.4 (5)	70.0 (21)	n.s.
Rituximab	56.8 (21)	57.1 (4)	56.7 (17)	n.s.
Methotrexate	27.0 (10)	28.6 (2)	26.7 (8)	n.s.
**Echocardiographic Parameters**				
LV GLS, %, mean (SD)	-17.9 (2.2)	-17.3 (2.4)	-18.0 (2.2)	n.s.
LV EF, %, mean (SD)	59.8 (4.1)	56.4 (2.2)	60.8 (4.0)	0.011
LV mass index, g/m², median (IQR)	75.0 (16.4)	74.0 (26.3)	76.5 (15.0)	n.s.
RV free wall strain, %, median (IQR)	-20.0 (10.7)	-20.0 (4.0)	-19.9 (5.2)	n.s.

IIM: idiopathic inflammatory myopathies; MSA: myositis-specific antibodies; CK: creatine kinase; CRP: C-reactive protein; ANA: antinuclear antibodies; LV: left ventricle; GLS: global longitudinal strain; EF: ejection fraction; RV: right ventricle; n.s.: not significant

### Clinical and laboratory correlates of GLS

To explore clinical and laboratory correlates of GLS, we performed univariate linear regression analyses. Both lymphocyte count at disease onset and the presence of arthritis were significantly associated with GLS. We then conducted multivariate linear regression including all variables listed in Table 2, selected based on their clinical and pathophysiological relevance in IIM. In the adjusted model, lymphocyte count, arthritis, and CK at disease onset remained independently associated with GLS values.

**Table 2:** Results of univariate and multivariate regression analyses exploring associations between GLS and selected clinical and laboratory parameters

Variable	UNIVARIATE		MULTIVARIATE	
	Beta (95% CI)	p	Beta (95% CI)	p
Age, years	0.023 (-0.051-0.096)	n.s.	0.032 (-0.030-0.094)	n.s.
Female sex	-0.700 (-1.500-0.100)	0.084	-	-
Lymphocytes at onset, /µL	0.001 (0.000-0.002)	0.042*	0.001 (0.000-0.002)	0.025*
Arthritis	1.781 (0.231–3.331)	0.026*	2.165 (0.610–3.719)	0.007**
CK at onset, U/L	0.0001 (-0.0002 to 0.0004)	0.396	0.0003 (0.0000 to 0.0005)	0.024*

CI: confidence interval, CK: creatine kinase; Higher beta values indicate worse (less negative) GLS; Multivariate model includes: lymphocytes, arthritis, CK, age (*N* = 31 with complete data). R² = 0.385, *p < 0.05, **p < 0.01, ***p < 0.001

To further investigate the role of arthritis, patients were stratified according to its presence (Table 3). Individuals with arthritis exhibited significantly worse GLS and lower CK levels, while EF remained comparable between groups. These findings consistently support an independent association between arthritis and impaired GLS, irrespective of EF.

**Table 3:** Comparison of clinical, laboratory, and echocardiographic characteristics of IIM patients according to the presence or absence of arthritis

Variable	Absence of arthritis (*n* = 14)	Presence of arthritis (*n* = 23)	*p*
**Demographics**			
Female sex, % (n)	71.4 (10)	87.0 (20)	0.461
Age, years, mean (SD)	64.6 (7.6)	64.4 (12.4)	0.955
**Laboratory parameters**			
Lymphocytes at onset, /µL, mean (SD)	1697 (771)	2095 (927)	0.222
CK at onset, U/L, median (IQR)	901 (492–4800)	302 (62–612)	**0.025**
ANA titer, median (IQR)	640 (320–960)	160 (80–320)	**0.022**
Echocardiographic parameters*			
LV GLS %, mean (SD)	-19.04 (2.04)	-17.14 (2.05)	**0.010**
EF %, mean (SD)	59.7 (5.0)	59.9 (3.6)	0.879

ANAs: antinuclear antibodies, CK: creatine kinase, LV GLS: left ventricle global longitudinal strain, EF: ejection fraction; **p < 0.05

## Discussion

### Speckle-Tracking echocardiography in clinical practice

Speckle-tracking echocardiography is an advanced imaging technique that analyzes myocardial deformation by tracking acoustic speckles frame-by-frame throughout the cardiac cycle. This technique offers several advantages for detecting subclinical cardiac dysfunction: (1) high feasibility with success rates > 90% in patients with adequate image quality; (2) good reproducibility with inter-observer variability < 10%; (3) incremental prognostic value over conventional parameters; and (4) potential to detect early myocardial involvement before EF decline. While cardiac magnetic resonance remains the gold standard for tissue characterization, GLS provides a readily available, cost-effective screening tool for subclinical dysfunction. A normal GLS value, defined as more negative than −18%, has a high negative predictive value for excluding significant myocardial involvement, making it valuable for routine cardiac assessment in IIM patients.

Recent meta-analyses have established reference ranges for GLS in healthy populations, with values more negative than − 18% generally considered normal [17–19]. Values between − 16% and − 18% represent a borderline range associated with increased cardiovascular risk, while values less negative than − 16% indicate significant dysfunction with prognostic implications [20]. Our IIM cohort’s mean GLS (-17.9%) falls within this borderline range, suggesting clinically relevant cardiac involvement that may warrant closer monitoring and follow-up.

Ejection fraction is a widely used global index of left ventricular function, reflecting the combined contribution of circumferential and longitudinal myocardial fibers. However, EF lacks the sensitivity to distinguish between these components and often remains within normal limits until significant myocardial dysfunction has occurred. Among the contractile components of the left ventricle, longitudinal function plays a crucial role by reducing the long-axis dimension of the LV cavity during systole, a motion primarily driven by the displacement of the mitral annulus toward the apex. This longitudinal shortening may account for up to 60% of the total stroke volume [21]. Longitudinal fibers, predominantly located in the subendocardial layer, are particularly vulnerable to a variety of pathological stimuli. This vulnerability is attributable to their high oxygen demand, greater exposure to wall stress, and relative proximity to inflammatory mediators [22, 23].

### Age-related effects and clinical significance

Although our IIM patients were older than the control group, the observed GLS impairment remained significant after adjusting for age and sex differences. This finding strengthens our conclusion that GLS reduction in IIM reflects disease-related myocardial involvement rather than normal aging effects. While age-related GLS decline has been reported [18], the GLS impairment observed in our IIM cohort, which remained significant after adjustment for age and sex (β = +2.25%), exceeds what would be expected from aging alone. Importantly, in our multivariate analysis within the IIM cohort, age was not significantly associated with GLS values, further supporting that the observed pattern remains consistent regardless of this demographic factor.

In our cohort, despite a preserved ejection fraction, the mean GLS was significantly below the lower limit of normality, indicating early and diffuse myocardial dysfunction. When compared with 37 healthy controls, this difference remained highly significant even after statistical correction for demographic differences (β = +2.25%, *p* < 0.001). This observation highlights the clinical utility of GLS in identifying subclinical cardiac involvement in IIM patients, which may otherwise go undetected when using conventional parameters such as EF [14–16]. In exploratory analyses, we did not observe a clear association between GLS impairment and specific IIM subtypes. However, this finding should be interpreted with caution, as the number of patients within each subtype was limited, potentially reducing the ability to detect meaningful subtype-specific differences in cardiac involvement.

### GLS in connective tissue diseases

Similar GLS impairment has been reported in other connective tissue diseases, particularly systemic sclerosis, where reduced GLS correlates with pulmonary arterial hypertension and predicts adverse outcomes. In systemic lupus erythematosus, subclinical LV dysfunction detected by GLS has been associated with disease activity and duration. These observations across different autoimmune conditions support the concept that systemic inflammation may share common pathways affecting myocardial function, and help contextualize our findings within the broader spectrum of connective tissue diseases.

Subclinical cardiac involvement detected through GLS, even in the absence of overt cardiovascular disease, has also been reported in other autoimmune and rheumatologic conditions [9, 24]. In particular, in patients with rheumatoid arthritis, impaired GLS has been shown to correlate with greater disease severity, increased morbidity, and higher mortality risk [9, 10, 25]. These findings reinforce the concept that systemic inflammation can affect myocardial function early in the disease course, making GLS a valuable non-invasive marker across different autoimmune settings.

### Systemic inflammation and cardiac dysfunction

Our findings support the concept that systemic inflammation plays a key role in early cardiac dysfunction in IIM. The association between lymphocyte count and GLS values, even when lymphocyte levels were within normal ranges, suggests that subtle immune activation may contribute to myocardial impairment. The relationship between inflammatory markers and cardiac dysfunction in autoimmune diseases has been increasingly recognized, with several studies demonstrating that subclinical inflammation can affect myocardial function before overt cardiovascular symptoms develop. In this context, the finding of worse GLS values in patients with concomitant arthritis may reflect a distinct disease phenotype characterized by greater systemic and extramuscular inflammatory involvement, rather than by the extent of primary muscle damage alone. This observation supports the hypothesis that subclinical myocardial dysfunction in IIM may be more closely related to systemic immune activation than to muscle enzyme levels.

Arthritis, although often mild and non-destructive, is a frequent extramuscular manifestation in IIM, particularly in patients with anti-Jo1 antibodies, and may precede the onset of muscle weakness [26–29]. Arthritis is also commonly observed during disease relapses [26]. In our cohort, the prevalence of arthritis was 62.2%, higher than reported in previous studies, where arthritis prevalence ranged between 25% and 40% [26, 27]. The higher prevalence observed in our cohort may reflect specific patient selection, potentially influenced by referral patterns, disease severity at presentation, or cohort characteristics. Given these observations, we included the presence of arthritis in our analyses, finding that it was independently associated with impaired GLS values. To further explore this association, we stratified patients based on the presence of arthritis, observing that those with arthritis had significantly worse GLS despite no significant difference in ejection fraction compared to patients without arthritis, as shown in Table 3. Interestingly, patients with arthritis also exhibited lower CK levels compared to those without arthritis. This observation might reflect a clinical phenotype characterized by predominant joint involvement and relatively reduced skeletal muscle inflammation, leading to lower serum CK values. Our findings are consistent with observations in patients with rheumatoid arthritis, where impaired GLS has been associated with greater disease severity, systemic inflammation, and poorer clinical outcomes [9–11, 13, 30–32].

### Limitations

This study has several limitations. First, the sample size was relatively small, which may limit the generalizability of our findings; nevertheless, considering the rarity of IIM, it can be considered acceptable for an exploratory investigation. Second, the retrospective cross-sectional design prevented us from evaluating the longitudinal evolution of GLS or the effects of treatment over time. Third, the control group was younger than our IIM patients, reflecting the typical age distribution in our echo database; however, statistical adjustment for age and sex confirmed that GLS impairment was independent of these demographic differences. Fourth, while we characterized patients by IIM subtype and myositis-specific antibody profile, we did not systematically apply standardized disease activity measures (MITAX/MDI), as the retrospective design limited the availability of comprehensive IMACS disease activity core set measures. Finally, we did not evaluate potential effects of immunosuppressive therapies on GLS values. While most patients were receiving treatment at the time of assessment (88.9% corticosteroids, 72.2% mycophenolate), the heterogeneous treatment regimens and retrospective design precluded systematic analysis of therapy effects on cardiac function.

## Conclusions

In patients with idiopathic inflammatory myopathies without known cardiovascular disease, we observed a global reduction in longitudinal systolic function as measured by GLS, despite preserved ejection fraction. This impairment remained highly significant even after adjusting for age and sex differences compared to healthy controls (β = +2.25%, *p* < 0.001), confirming that the observed dysfunction reflects disease-related cardiac involvement rather than normal aging effects. Our findings suggest that GLS may serve as a sensitive marker for detecting early, subclinical myocardial involvement in this population. Moreover, we identified that the presence of arthritis, along with higher lymphocyte counts at disease onset, was independently associated with impaired GLS. This suggests that systemic inflammatory activity may play a pivotal role in early cardiac dysfunction among patients with IIM. Routine integration of GLS in the echocardiographic evaluation of IIM patients may assist in the early detection of cardiac involvement, even in asymptomatic individuals. Given the subclinical cardiac involvement demonstrated in our cohort, longitudinal studies are warranted to evaluate the evolution of GLS over time and to clarify its potential role in guiding clinical management and therapeutic strategies.

## Data Availability

No datasets were generated or analysed during the current study.
