# Correction: Subclinical cardiac dysfunction in idiopathic inflammatory myopathies: the role of global longitudinal strain

**DOI:** 10.1007/s10238-026-02123-5

**Published:** 2026-03-30

**Authors:** Simone Romano, Andrea Sartorio, Chiara Dal Pont, Francesca Segatta, Marta Piazzola, Marco Vicardi, Mattia Cominacini, Federico Aldegheri, Riccardo Bixio, Ombretta Viapiana

**Affiliations:** 1 Department of Internal Medicine, Section of Internal Medicine C, University of Verona, Piazzale Scuro 37134, Verona, Italy; 2 Rheumatology Unit, University of Verona, Verona, Italy

**Correction to: Clinical and Experimental Medicine (2026) 26:158**

https://doi.org/10.1007/s10238-026-02054-1

In the original version of the article, in Abstract section seventh sentence which previously read as

In linear regression models, GLS was independently associated with arthritis (β = 2.165, p = 0.007), lymphocyte count (β = 0.001, p = 0.025), CK levels (β = 0.000, p = 0.024), and age (β = 0.032, p = 0.020).

But should have been

In linear regression models, GLS was independently associated with arthritis (β = 2.165, p = 0.007), lymphocyte count (β = 0.001, p = 0.025), CK levels (β = 0.0003, p = 0.024).

In Methods Section, first subdivision which previously read as Study designs and participants but should have been Study design and participants.

Similarly, in Results Section, first subdivision which previously read as Patients characteristics but should have been Patient characteristics

Tables 1 and 2 which previously read as

**Table 1:** Patient characteristics of the IIM cohort, including sex-based comparisons

Variable	Total (*n* = 37)	Male (*n* = 7)	Female (*n* = 30)	*p*
**Demographics**				
Age, years, mean (SD)	63.8 (10.6)	61.1 (8.3)	64.4 (11.1)	n.s.
Age of onset, years, mean (SD)	54.9 (11.7)	54.6 (9.2)	54.9 (12.3)	n.s.
Weight, kg, mean (SD)	70.0 (12.2)	83.2 (13.4)	67.3 (10.3)	0.006
Time to therapy, months, median (IQR)	3 (4)	3.5 (2.5)	2 (4)	n.s.
**IIM Subtypes**,** % (n)**				
Dermatomyositis	40.5 (15)	28.6 (2)	43.3 (13)	n.s.
Anti-synthetase syndrome	29.7 (11)	42.9 (3)	26.7 (8)	n.s.
Polymyositis	16.2 (6)	14.3 (1)	16.7 (5)	n.s.
Necrotizing/Overlap myositis	13.5 (5)	14.3 (1)	13.3 (4)	n.s.
**Myositis-Specific Antibodies**,** % (n)**				
Any MSA positive	86.5 (32/37)	85.7 (6/7)	86.7 (26/30)	n.s.
Anti-Jo1	29.7 (11/37)	42.9 (3/7)	26.7 (8/30)	n.s.
Anti-Ro52	32.4 (12/37)	28.6 (2/7)	33.3 (10/30)	n.s.
Anti-Mi2	8.1 (3/37)	0 (0/7)	10.0 (3/30)	n.s.
Anti-SRP	5.4 (2/37)	14.3 (1/7)	3.3 (1/30)	n.s.
Other MSAs	10.8 (4/37)	0 (0/7)	13.3 (4/30)	n.s.
**Laboratory Parameters**				
Lymphocytes at onset, mean (SD) [µL normal range: 1000–4000]	1945 (881)	2370 (1077)	1848 (823)	n.s.
CK at onset, median (IQR) [U/L, normal: F < 145, M < 171]	492 (1112)	843 (789)	461 (1298)	n.s.
CRP at onset, median (IQR) [mg/L, normal range]	3.0 (8.0)	4.0 (2.5)	3.0 (4.0)	n.s.
ANA titer, median (IQR)	160 (480)	320 (0)	160 (640)	n.s.
**Clinical Manifestations**,** % (n)**				
Arthritis	62.2 (23)	42.9 (3)	66.7 (20)	n.s.
Skin manifestations	40.5 (15)	42.9 (3)	40.0 (12)	n.s.
Interstitial lung disease	40.5 (15)	71.4 (5)	33.3 (10)	0.087
Dysphagia	21.6 (8)	14.3 (1)	23.3 (7)	n.s.
Gastrointestinal symptoms	24.3 (9)	14.3 (1)	26.7 (8)	n.s.
Pulmonary arterial hypertension	2.7 (1)	14.3 (1)	0 (0)	n.s.
**Current Immunosuppressive Therapy**,** % (n)**				
Corticosteroids	89.2 (33)	85.7 (6)	90.0 (27)	n.s.
Mycophenolate mofetil	70.3 (26)	71.4 (5)	70.0 (21)	n.s.
Rituximab	56.8 (21)	57.1 (4)	56.7 (17)	n.s.
Methotrexate	27.0 (10)	28.6 (2)	26.7 (8)	n.s.
**Echocardiographic Parameters**				
LV GLS, %, mean (SD)	-17.9 (2.2)	-17.3 (2.4)	-18.0 (2.2)	n.s.
LV EF, %, mean (SD)	59.8 (4.1)	56.4 (2.2)	60.8 (4.0)	0.011
LV mass index, g/m², median (IQR)	75.0 (16.4)	74.0 (26.3)	76.5 (15.0)	n.s.
RV free wall strain, %, median (IQR)	-20.0 (10.7)	-20.0 (4.0)	-19.9 (5.2)	n.s.

IIM: idiopathic inflammatory myopathies; MSA: myositis-specific antibodies; CK: creatine kinase; CRP: C-reactive protein; ANA: antinuclear antibodies; LV: left ventricle; GLS: global longitudinal strain; EF: ejection fraction; RV: right ventricle; n.s.: not significant

**Table 2:** Results of univariate and multivariate regression analyses exploring associations between GLS and selected clinical and laboratory parameters

Variable	UNIVARIATE		MULTIVARIATE	
	Beta (95% CI)	p	Beta (95% CI)	p
Age, years	0.023 (-0.051-0.096)	n.s.	0.032 (-0.030-0.094)	n.s.
Female sex	-0.700 (-1.500-0.100)	0.084	-	-
Lymphocytes at onset, /µL	0.001 (0.000-0.002)	0.042*	0.001 (0.000-0.002)	0.035*
Arthritis	1.781 (0.231–3.331)	0.026*	2.165 (0.610–3.719)	0.008**
CK at onset, U/L	0.0001 (-0.0002 to 0.0004)	0.396	0.0003 (0.0000 to 0.0005)	0.030*

CI: confidence interval, CK: creatine kinase; Higher beta values indicate worse (less negative) GLS; Multivariate model includes: lymphocytes, arthritis, CK, age (*N* = 31 with complete data). R² = 0.385, *p < 0.05, **p < 0.01, ***p < 0.001

But should have been

**Table 1:** Patient characteristics of the IIM cohort, including sex-based comparisons

Variable	Total (*n* = 37)	Male (*n* = 7)	Female (*n* = 30)	*p*
**Demographics**				
Age, years, mean (SD)	63.8 (10.6)	61.1 (8.3)	64.4 (11.1)	n.s.
Age of onset, years, mean (SD)	54.9 (11.7)	54.6 (9.2)	54.9 (12.3)	n.s.
Weight, kg, mean (SD)	70.0 (12.2)	83.2 (13.4)	67.3 (10.3)	0.006
Time to therapy, months, median (IQR)	3 (4)	3.5 (2.5)	2 (4)	n.s.
**IIM Subtypes**,** % (n)**				
Dermatomyositis	40.5 (15)	28.6 (2)	43.3 (13)	n.s.
Anti-synthetase syndrome	29.7 (11)	42.9 (3)	26.7 (8)	n.s.
Polymyositis	16.2 (6)	14.3 (1)	16.7 (5)	n.s.
Necrotizing/Overlap myositis	13.5 (5)	14.3 (1)	13.3 (4)	n.s.
**Myositis-Specific Antibodies**,** % (n)**				
Any MSA positive	86.5 (32/37)	85.7 (6/7)	86.7 (26/30)	n.s.
Anti-Jo1	29.7 (11/37)	42.9 (3/7)	26.7 (8/30)	n.s.
Anti-Ro52	32.4 (12/37)	28.6 (2/7)	33.3 (10/30)	n.s.
Anti-Mi2	8.1 (3/37)	0 (0/7)	10.0 (3/30)	n.s.
Anti-SRP	5.4 (2/37)	14.3 (1/7)	3.3 (1/30)	n.s.
Other MSAs	10.8 (4/37)	0 (0/7)	13.3 (4/30)	n.s.
**Laboratory Parameters**				
Lymphocytes at onset, mean (SD) [µL normal range: 1000–4000]	1945 (881)	2370 (1077)	1848 (823)	n.s.
CK at onset, median (IQR) [U/L, normal: F < 145, M < 171]	492 (1112)	843 (789)	461 (1298)	n.s.
CRP at onset, median (IQR) [mg/L, normal range]	3.0 (8.0)	4.0 (2.5)	3.0 (4.0)	n.s.
ANA titer, median (IQR)	160 (480)	320 (0)	160 (640)	n.s.
**Clinical Manifestations**,** % (n)**				
Arthritis	62.2 (23)	42.9 (3)	66.7 (20)	n.s.
Skin manifestations	40.5 (15)	42.9 (3)	40.0 (12)	n.s.
Interstitial lung disease	40.5 (15)	71.4 (5)	33.3 (10)	n.s.
Dysphagia	21.6 (8)	14.3 (1)	23.3 (7)	n.s.
Gastrointestinal symptoms	24.3 (9)	14.3 (1)	26.7 (8)	n.s.
Pulmonary arterial hypertension	2.7 (1)	14.3 (1)	0 (0)	n.s.
**Current Immunosuppressive Therapy**,** % (n)**				
Corticosteroids	89.2 (33)	85.7 (6)	90.0 (27)	n.s.
Mycophenolate mofetil	70.3 (26)	71.4 (5)	70.0 (21)	n.s.
Rituximab	56.8 (21)	57.1 (4)	56.7 (17)	n.s.
Methotrexate	27.0 (10)	28.6 (2)	26.7 (8)	n.s.
**Echocardiographic Parameters**				
LV GLS, %, mean (SD)	-17.9 (2.2)	-17.3 (2.4)	-18.0 (2.2)	n.s.
LV EF, %, mean (SD)	59.8 (4.1)	56.4 (2.2)	60.8 (4.0)	0.011
LV mass index, g/m², median (IQR)	75.0 (16.4)	74.0 (26.3)	76.5 (15.0)	n.s.
RV free wall strain, %, median (IQR)	-20.0 (10.7)	-20.0 (4.0)	-19.9 (5.2)	n.s.

IIM: idiopathic inflammatory myopathies; MSA: myositis-specific antibodies; CK: creatine kinase; CRP: C-reactive protein; ANA: antinuclear antibodies; LV: left ventricle; GLS: global longitudinal strain; EF: ejection fraction; RV: right ventricle; n.s.: not significant

**Table 2:** Results of univariate and multivariate regression analyses exploring associations between GLS and selected clinical and laboratory parameters

Variable	UNIVARIATE		MULTIVARIATE	
	Beta (95% CI)	p	Beta (95% CI)	p
Age, years	0.023 (-0.051-0.096)	n.s.	0.032 (-0.030-0.094)	n.s.
Female sex	-0.700 (-1.500-0.100)	0.084	-	-
Lymphocytes at onset, /µL	0.001 (0.000-0.002)	0.042*	0.001 (0.000-0.002)	0.025*
Arthritis	1.781 (0.231–3.331)	0.026*	2.165 (0.610–3.719)	0.007**
CK at onset, U/L	0.0001 (-0.0002 to 0.0004)	0.396	0.0003 (0.0000 to 0.0005)	0.024*

CI: confidence interval, CK: creatine kinase; Higher beta values indicate worse (less negative) GLS; Multivariate model includes: lymphocytes, arthritis, CK, age (*N* = 31 with complete data). R² = 0.385, *p < 0.05, **p < 0.01, ***p < 0.001

In Discussion section, second subdivision which previously read as Age-Related effects and clinical significance but should have been Age-related effects and clinical significance.

In reference section the last reference Nowell M, Fine Cynthia S, Crowson Grace, Lin Jae K, Oh Hector R, Villarraga Sherine E, Gabriel. Evaluation of myocardial function in patients with rheumatoid arthritis using strain imaging by speckle-tracking echocardiography. Ann Rheum Dis. 2014;73(10):1833–39. https://doi.org/10.1136/annrheumdis—2013—203314 has been,mistakenly included.

The original version of the article has been corrected.

